# Trilobatin, a Natural Food Additive, Exerts Anti-Type 2 Diabetes Effect Mediated by Nrf2/ARE and IRS-1/GLUT2 Signaling Pathways

**DOI:** 10.3389/fphar.2022.828473

**Published:** 2022-01-27

**Authors:** Yan-Ling Shi, Yue-Ping Zhang, Huan Luo, Fan Xu, Jian-Mei Gao, Jing-Shan Shi, Qi-Hai Gong

**Affiliations:** ^1^ Key Laboratory of Basic Pharmacology of Ministry of Education and Joint International Research Laboratory of Ethnomedicine of Ministry of Education, Zunyi Medical University, Zunyi, China; ^2^ Key Laboratory of Basic Pharmacology of Guizhou Province, Zunyi Medical University, Zunyi, China; ^3^ Spemann Graduate School of Biology and Medicine (SGBM), Albert-Ludwigs-University Freiburg, Freiburg, Germany; ^4^ Department of Pharmacology, School of Pharmacy, Zunyi Medical University, Zunyi, China

**Keywords:** trilobatin, T2DM, insulin resistance, lipid metabolism, Nrf2/ARE signaling pathway, insulin signaling transduction pathway

## Abstract

Oxidative stress and aberrant insulin signaling transduction play vital roles in type 2 diabetes mellitus (T2DM). Our previous research has demonstrated that trilobatin (TLB), derived from the leaves of *Lithocarpus Polystachyus* (Wall.), exhibits a potent antioxidative profile. In the current study, we investigated the anti-T2DM effect of TLB on KK-Ay diabetic mice and further explored the potential mechanisms. Our results showed that TLB significantly reduced the high fasting blood glucose level and insulin resistance and promoted the tolerances to exogenous glucose and insulin in KK-Ay mice. Moreover, TLB reduced the content of reactive oxygen species; enhanced antioxidant enzymes activities, including serum catalase, glutathione peroxidase, and superoxide dismutase; and regulated the abnormal parameters of lipid metabolism, including triglyceride, high-density lipoprotein-cholesterol, low-density lipoprotein-cholesterol, and free fatty acid, as evidenced by enzyme-linked immunosorbent assay. Additionally, TLB markedly ameliorated the pancreatic islet morphology near normal and increased the insulin expression of the islet. Whereafter, TLB promoted Nrf2 that was translocated from cytoplasm to nucleus. Moreover, it increased the protein expressions of HO-1, NQO-1, and GLUT-2, and phosphorylation levels of Akt and GSK-3*β* ^Ser 9^ and decreased the protein expressions of keap1 and phosphorylation levels of IRS-1^Ser 307^ and GSK-3*β* ^Tyr 216^. Taken together, our findings reveal that TLB exhibits an anti-T2DM effect in KK-Ay mice by activating the Nrf2/ARE signaling pathway and regulating insulin signaling transduction pathway, and TLB is promising to be developed into a novel candidate for the treatment of T2DM in clinic due to its favorable druggability.

## Introduction

There were about 537 million adult diabetes mellitus (DM) patients worldwide in 2021, among which type 2 diabetes mellitus (T2DM) patients accounted for approximately 90% (IDF Diabetes Atlas 10th edition, 2021). Due to its high morbidity, T2DM imposes a heavy social and economic burden on individuals and society. With the progression of T2DM, patients suffer from hyperglycemia, dyslipidemia, and insulin resistance, as well as devastating complications, including diabetic neuropathy, diabetic retinopathy, and diabetic nephropathy (Galicia-Garcia et al., 2020). Currently, multiple hypoglycemic drugs are widely applied to treat T2DM, such as sulfonylureas, biguanides, thiazolidinediones, sodium-glucose cotransporter-2 inhibitors, glucagon-like peptide 1 receptor agonists, and dipeptidyl peptidase-4 inhibitors (Vaughan et al., 2020; Taylor et al., 2021). In contrast, the clinical application of the above-mentioned drugs is tremendously limited due to a series of severe adverse drug reactions or single target of action (Vijayakumar et al., 2017; Rajasurya et al., 2019). Hence, safe and cost-efficient anti-T2DM drugs are desperately needed in clinic.

To date, the exact pathogenesis of T2DM has not been fully elucidated. Emerging evidence shows that oxidative stress plays a vital role in T2DM (Contrepois et al., 2020). Patients with T2DM generate abundant highly active molecules such as reactive oxygen species (ROS), which lead to excessive oxidative stress and damage internal biological macromolecules, eventually aggravating T2DM or diabetic complications (Antonetti et al., 2021). Moreover, oxidative stress ulterior injures islet *β* cells, consequently perturbs insulin synthesis and secretion. Oxidative stress stimuli trigger dysfunction of insulin-stimulated glucose transport and the insulin signaling transduction pathway, which subsequently causes insulin resistance (Apostolova et al., 2020). Therefore, alleviation of oxidative stress and suppression of aberrant insulin signaling transduction are promising therapeutic tactics of T2DM.

**FIGURE 1:**
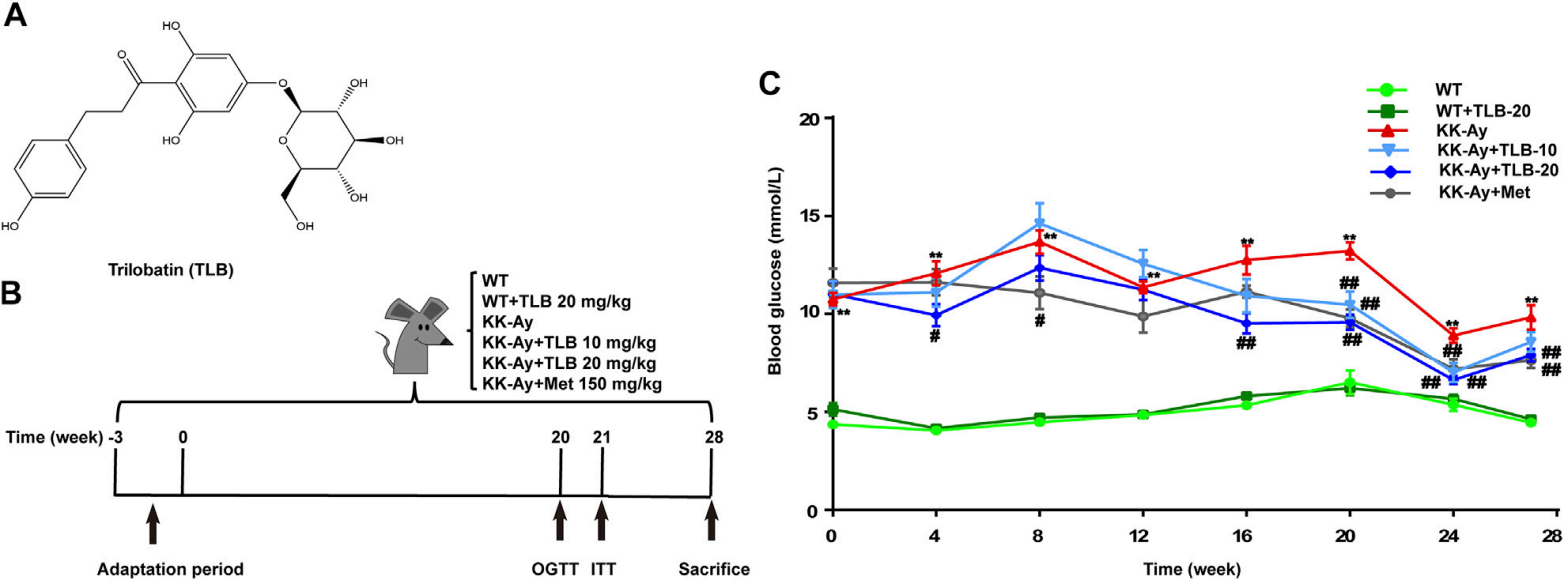
TLB reduced high blood glucose level in KK-Ay mice. **(A)** Chemical structure of TLB. **(B)** Schematic diagram of the experimental protocols. **(C)** The FBG curve of every group. ^**^ *p* < .01 *vs.* WT group; ^*#*^ *p* < .05*,* ^*##*^ *p* < .01 *vs.* KK-Ay group; *n* = 8–12 per group, mean ± SEM.

Emerging studies have proven that bioactive components derived from natural plants exhibit potent therapeutic contributions to T2DM medical treatment (Unuofin and Lebelo, 2020). For instance, metformin (Met) is the first-line drug for treating T2DM, deriving from galegine, which is the major active ingredient of European goat’s rue *Galega officinalis* L. (Hunter et al., 2018). Trilobatin (TLB) is a natural dihydrochalcone small molecule from the leaves of *Lithocarpus Polystachyus* (Wall.) Rehd. utilized as folk medicine to treat multiple diseases (Figure 1A) (Shang et al., 2020). Of note, TLB is also used as a natural food additive with high sweetness but low calories and displays an anti-DM effect on high-fat diet (HFD) or streptozotocin-induced DM mice (Wang et al., 2016). Moreover, it also improves insulin resistance in ob/ob mice by activating the insulin receptor substrate (IRS)/protein kinase B (Akt)/glucose transporter-4 (GLUT-4) signaling pathway (Liu et al., 2020). Interestingly, our previous studies demonstrated that TLB exerts predominant neuroprotective effects due to its striking antioxidative profile (Gao et al., 2020; Li et al., 2021). However, whether TLB can exert an anti-T2DM effect and the possible mechanism remain undiscovered. Therefore, the current study was designed to investigate the effect of TLB on T2DM in KK-Ay mice, a model animal of T2DM, and further explore its underlying mechanisms.

## Materials and Methods

### Animals and Agents

Male KK-Ay mice (8 weeks) and C57/BL6J mice (7 weeks) were purchased from Beijing Huafukang Biotechnology Co., Ltd. (Beijing, China; Certificate no. SCXK 2019-0008), housed at 23 ± 1°C specific-pathogen-free (SPF) environment with a 12 h light/dark cycle and 60 ± 2% humidity. The KK-Ay mice were fed with HFD from Beijing Huafukang Biotechnology Co., Ltd. (Beijing, China; Certificate no. SCXK 2019-0008), and the C57 mice were fed with a standard chow diet. Diet and tap water were provided *ad libitum*. TLB (purity ≥98% by HPLC, Lot no. 19041605) and Met were purchased from Chengdu Push Bio-Technology Medical Technology Corporation (Sichuan, China) and Sino-American Shanghai Squibb Pharmaceuticals Ltd. (Shanghai, China). Both were dissolved in normal saline (NS) using ultrasonication for 30 min. Glucometer and matching test paper were obtained from Roche Diabetes Care Inc. (Basel, Switzerland). An immunohistochemical staining kit was obtained from Beijing Zhongshan Jinqiao Biological Technology Co., Ltd. (Beijing, China). Commercial enzyme-linked immunosorbent assay (ELISA) kits of ROS, glutathione peroxidase (GSH-Px), catalase (CAT), superoxide dismutase (SOD), fasting insulin (FINS), triglyceride (TG), high-density lipoprotein-cholesterol (HDL-C), low-density lipoprotein-cholesterol (LDL-C), and free fatty acid (FFA) were purchased from Shanghai Renjie Biotechnology Co., Ltd. (Shanghai, China). Primary antibodies including Kelch-like ECH-associated protein 1 (keap1) (ab227828), Nrf2 (ab31163), hemeoxygenase-1(HO-1) (ab189491), NAD(P)H-quinone oxidoreductase 1 (NQO-1) (ab34173), insulin (ab181547), insulin receptor (IR) (ab203746), p-IR^Tyr 1185^ (ab203278), IRS-1 (ab52167), p-IRS-1^Ser 307^ (ab5599), Akt (ab179463), p-Akt (ab131443), glycogen synthase kinase (GSK)-3*β* (ab93926), p-GSK-3β^Tyr 216^ (ab75745), p-GSK-3*β* ^Ser 9^ (ab131097), GLUT-2 (ab54460), *β*-tubulin (ab179513), and proliferating cell nuclear antigen (PCNA) (ab29) were purchased from Abcam Inc. (Cambridge, United States). Anti-*β*-actin (66009-1-1 g) and secondary antibodies were obtained from Proteintech Group, Inc. (Rosemont, IL, United States).

### TLB Administration and Grouping

In brief, after 3 weeks of adaptive feeding, blood samples were collected from tail veins of KK-Ay mice after fasting for 12 h, and the fasting blood glucose (FBG) was tested using a glucometer. FBG ≥7.0 mmol/L of KK-Ay mice were accepted in the following experiment. Afterward, the T2DM mice were randomly divided into four groups: NS-treated group (KK-Ay), TLB-treated at 10 and 20 mg/kg groups (KK-Ay + TLB-10, KK-Ay + TLB-20), and Met-treated at 150 mg/kg group (KK-Ay + Met). Accordingly, the wild-type (WT) mice were randomly divided into NS-treated group (WT) and TLB-treated at 20 mg/kg group (WT + TLB-20). The mice of the TLB-treated groups or Met-treated group were administered intragastrically TLB or Met. In contrast, the mice of the NS-treated groups were administered intragastrically volume-matched saline. All groups were received gavage administration twice a day for 28 weeks (Figure 1B).

### FBG Test

Briefly, the FBG of mice was tested regularly each week for 28 weeks. After fasting for 12 h, the blood samples were obtained from tail veins of WT mice or KK-Ay mice, and the FBG was detected by a glucometer.

### Oral Glucose Tolerance Test

To detect the ability of the glycemic regulatory in mice, the OGTT was performed on the 20th week after treatment with TLB or Met. In brief, mice were orally administrated with 2 g/kg glucose, which was dissolved in double-distilled water after fasting for 12 h. The blood samples were collected and measured at 0, 30, 60, 90, and 120 min after glucose loading, and then the area under the curve (AUC) of blood glucose was calculated as described in a previous study (Chen et al., 2018).

### Insulin Tolerance Test

The ITT was implemented to test the sensitivity of mice to exogenous insulin on the 21st week after treatment with TLB or Met. Hence, after 12 h of fasting, ITT was carried out in mice by intraperitoneal injection of recombinant human insulin, which was dissolved in NS. The blood samples were collected and measured at different time points (0, 30, 60, 90, and 120 min) after insulin injection, followed by calculating the AUC of blood glucose as mentioned in a previous study (Liu et al., 2017a).

### ELISA

The oxidative stress and lipid metabolism indicators were determined using ELISA assay. Briefly, by the end of drug treatment, blood samples of mice were obtained and centrifugated at 1,123 *×g* for 15 min at 4°C. Then, lipid metabolism indicators (TG, HDL-C, LDL-C, and FFA), antioxidant enzymes (GSH-Px, CAT, and SOD), ROS, and FINS were measured using commercial ELISA kits according to the manufacturer’s instruction.

### HOMA-IR

To quantify insulin resistance degree in mice, serum FINS levels were detected by the commercial ELISA kit according to the manufacturer’s protocol and then combined with the FBG to calculate HOMA-IR as described previously (Zhang et al., 2020).

### Hematoxylin and Eosin (H&E) Staining

In brief, pancreases were isolated, dehydrated, and embedded into paraffin after being fixed in a 10% neutral formalin solution at 4°C for 48 h. Subsequently, the paraffin sections of the pancreas (3.5 μm thicknesses) were stained with H&E, and then the histopathology was observed using an optical microscope.

### Immunohistochemical Staining

Likewise, pancreases were fixed with a 10% neutral formalin solution, dehydrated, and embedded in paraffin. Subsequently, the slices of the pancreas (3.5 μm thicknesses) were dewaxed with xylene and ethanol and incubated with 3% H_2_O_2_ in the dark at 37°C for 15 min. Thereafter, the sections were subjected to blocking with goat serum for 30 min at 37°C and then cultured with primary antibody against insulin (1:10,000) for 18 h at 4°C. Then, the slices were incubated with secondary antibody and HRP-labeled streptozotocin at 37°C for 20 min. Finally, the DAB coloration kit was used to mark insulin; the images were taken by optical microscope and analyzed using Image-Pro Plus software.

### Western Blot Analysis

In brief, 40–50 mg of −80°C frozen livers and pancreases was lysed by RIPA buffer with protease inhibitors and protein phosphatase inhibitors. After 13201 *×g* centrifugation at 4°C for 15 min, the supernatants were carefully extracted and quantified by BCA assay. Subsequently, equal amounts of protein samples (30 μg) were electrophoresed and separated on 8–10% sodium dodecylsulfate-polyacrylamide gel electrophoresis and transferred onto the membranes of polyvinylidene fluoride. Then, the membranes were blocked with 5% non-fat milk for 2 h at room temperature. Thereafter, membranes were incubated with a series of primary antibodies including anti-keap1 (1:1,000), anti-Nrf2 (1:1,000), anti-HO-1 (1:1,000), anti-NQO-1 (1:1,000), anti-IR (1:500), anti-p-IR^Tyr 1185^ (1:500), anti-IRS-1 (1:500), anti-p-IRS-1^Ser 307^ (1:500), anti-Akt (1:1,000), anti-p-Akt (1:1,000), anti-GSK-3β (1:1,000), anti-p-GSK-3β^Tyr 216^ (1:1,000), anti-p-GSK-3*β* ^Ser 9^ (1:1,000), anti-GLUT-2 (1:1,000), anti-*β*-tubulin (1:1,000), anti-PCNA (1:1,000), and anti-*β*-actin (1:2,000) dissolved in Tris-buffered saline containing 0.1% Tween-20 (TBS-T) with bovine serum albumin at 4°C for 17–18 h. Ultimately, incubated membranes with secondary antibodies at 4°C for 45–50 min, dropped ECL luminescent solution to form the images, and used ChemiDoc MP Imaging System (Bio-Rad Laboratories, Inc., Hercules, CA, United States) to quantify the band optical intensity.

### Statistical Analysis

All data are expressed as mean ± standard error (SEM) and analyzed using SPSS 18.0 (SPSS, Inc., Chicago, United States). Statistical significance between groups was analyzed by the general linear model of univariate ANOVA followed by the Bonferroni *post hoc* test. Differences with *p* < .05 were considered statistically significant.

## Results

### TLB Decreased High Blood Glucose Level in KK-Ay Mice

To investigate whether TLB could decrease the high blood glucose level of KK-Ay mice, the FBG was tested. The results showed that FBG of KK-Ay mice was dramatically increased more than that of the WT mice. However, FBG of mice in the KK-Ay + TLB-10 group at the time points of 20 and 24 weeks and the KK-Ay + TLB-20 group at the different time points (4, 16, 20, 24, and 28 weeks) were markedly decreased compared with the KK-Ay group (Figure 1C). Of note, TLB alone did not affect the FBG of WT mice. These findings suggested that TLB significantly reduced the blood glucose level with safety profile.

### TLB Improved Glucose Tolerance and Insulin Tolerance of KK-Ay Mice

**FIGURE 2:**
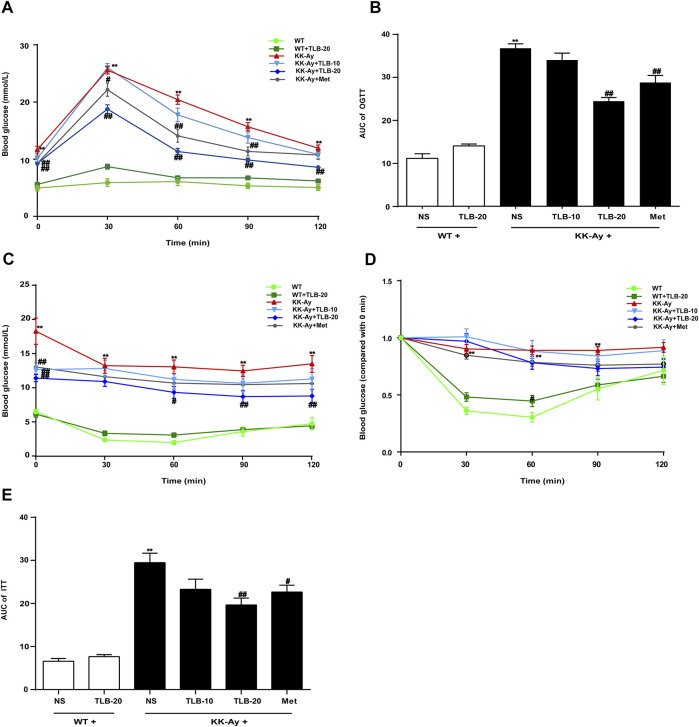
TLB improved glucose tolerance and insulin tolerance of KK-Ay mice. **(A)** The blood glucose curve of OGTT. **(B)** The AUC of OGTT. **(C)** The blood glucose curve of ITT. **(D)** The percentage of blood glucose (compared with 0 min) curve of ITT. **(E)** The AUC of ITT.^**^ *p* < .01 *vs.* WT group; ^*#*^ *p* < .05, ^*##*^ *p* < .01 *vs.* KK-Ay group; *n* = 9–10 per group, mean ± SEM.

To evaluate the effects of TLB on glucose tolerance and insulin tolerance in KK-Ay mice, OGTT and ITT were performed at the time points of 20 and 21 weeks, respectively. The blood glucose curve of OGTT showed that the blood glucose level of KK-Ay mice was markedly increased more than that of WT mice at different time points (0, 30, 60, 90, 120 min) after 2 g/kg glucose administration. In contrast, the blood glucose levels of mice in the KK-Ay + TLB-20 group were markedly decreased compared to that of the KK-Ay group at different time points (Figure 2A). Likewise, the AUC of blood glucose of KK-Ay mice was significantly exceeded compared to that of WT mice. However, the AUC of blood glucose in the KK-Ay + TLB-20 group was markedly decreased compared to that of the KK-Ay group (Figure 2B). Similarly, at 0, 30, 60, 90, and 120 min after administration of 0.75 U/kg recombinant human insulin, the blood glucose level and its AUC of KK-Ay mice were markedly increased compared to those of WT mice. However, the blood glucose level of mice in the KK-Ay + TLB-20 group at 0, 60, 90, and 120 min time points and its AUC were markedly reduced compared with the KK-Ay group (Figures 2C–E). These findings illustrated that TLB exerted a beneficial effect on improving glucose tolerance and insulin tolerance in KK-Ay mice.

### TLB Suppressed Insulin Resistance in KK-Ay Mice

**FIGURE 3:**
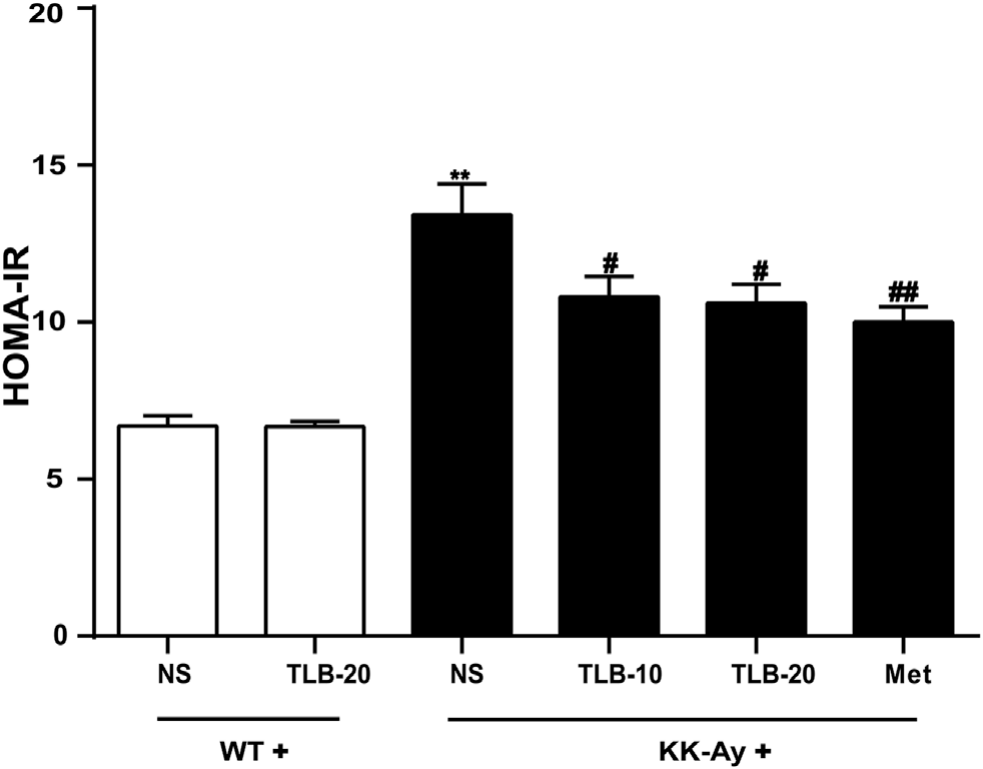
TLB suppressed insulin resistance in KK-Ay mice. ^**^ *p* < .01 *vs*. WT group; ^*#*^ *p* < .05, ^*##*^ *p* < .01 *vs*. KK-Ay group; *n* = 8–10 per group, mean ± SEM.

To evaluate the extent of insulin resistance in KK-Ay mice, HOMA-IR was determined according to the specific formula. The HOMA-IR value of KK-Ay mice was dramatically increased more than that of WT mice. However, the HOMA-IR values of mice in the KK-Ay + TLB-10 group and KK-Ay + TLB-20 group were markedly decreased compared to that of the KK-Ay group (Figure 3), which suggested that TLB effectively suppressed insulin resistance in KK-Ay mice.

### TLB Ameliorated Islet Morphology and Hypoinsulinemia in KK-Ay Mice

**FIGURE 4:**
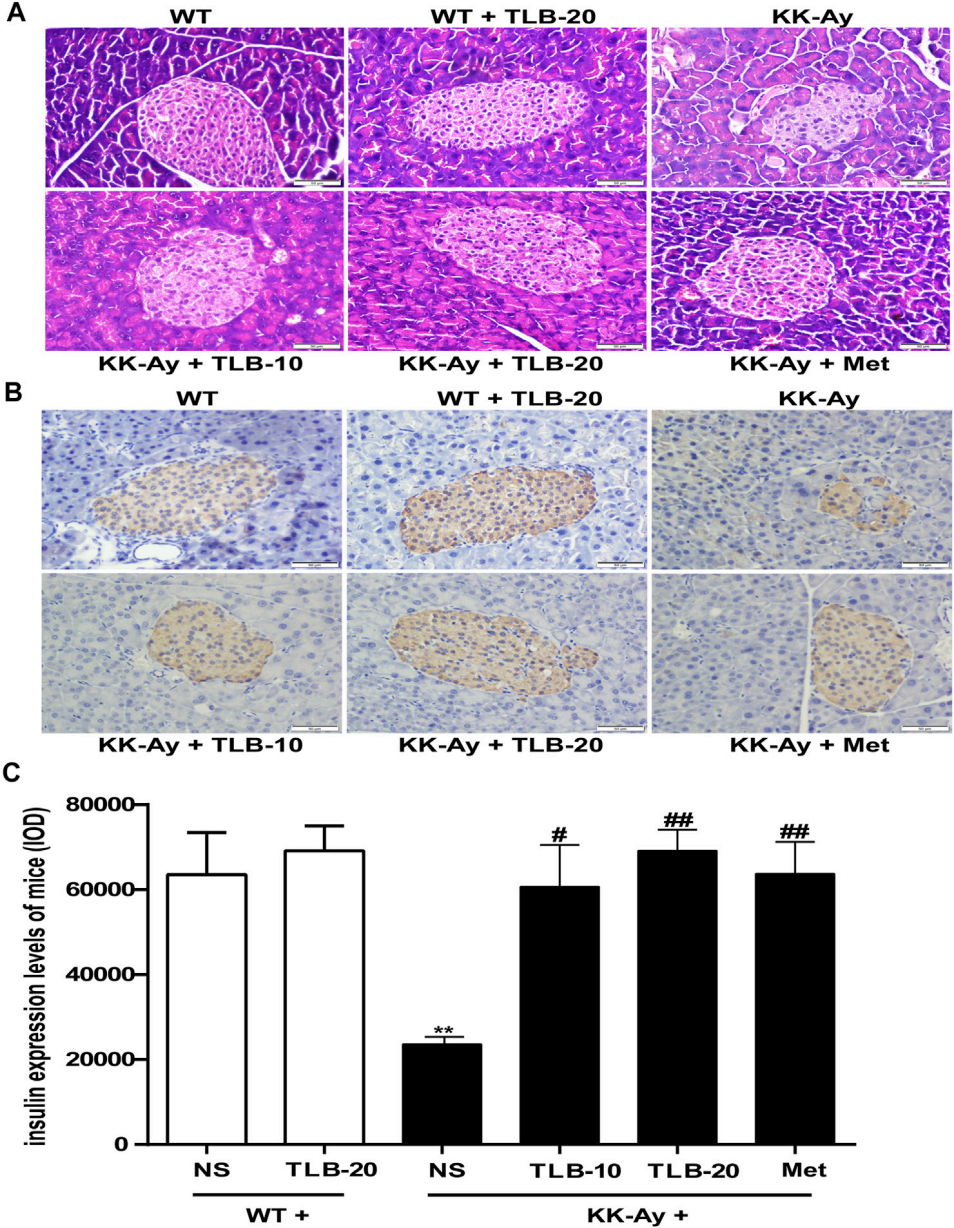
TLB ameliorated islets morphology and hypoinsulinemia in KK-Ay mice. **(A)** Representative images of H and E staining of pancreases (×400, scale bar = 50 μm). **(B)** Representative images of IHC. **(C)** Quantification of insulin expression by using Image-Pro Plus. ^**^ *p* < .01 *vs.* WT group; ^*#*^ *p* < .05, ^*##*^ *p* < .01 *vs.* KK-Ay group; *n* = 4 per group, mean ± SEM.

To explore whether TLB could protect pancreatic islets and improve hypoinsulinemia in the late phase of T2DM in KK-Ay mice, the islet morphology and insulin expression were observed by H&E staining and immunohistochemical staining. The results demonstrated that the islets of KK-Ay mice were destroyed as evidenced that the volume was significantly reduced, the morphology appeared uneven, and the number of cells in the islets was dramatically decreased compared to those of WT mice. In contrast, TLB markedly reversed these changes of pathological pancreatic islets in KK-Ay mice (Figure 4A). Similar to the results of H&E staining, the insulin expression of KK-Ay mice was dramatically decreased compared to that of WT mice. However, the insulin expression of mice in the KK-Ay + TLB-10 group and KK-Ay + TLB-20 group was markedly increased more than that of the KK-Ay group, as evidenced by immunohistochemical staining (Figures 4B,C). According to the above results, TLB effectively retrieved the islets damage and increased the secretion of insulin of KK-Ay mice.

### TLB Alleviated Lipid Metabolism Disorder in KK-Ay Mice

**FIGURE 5:**
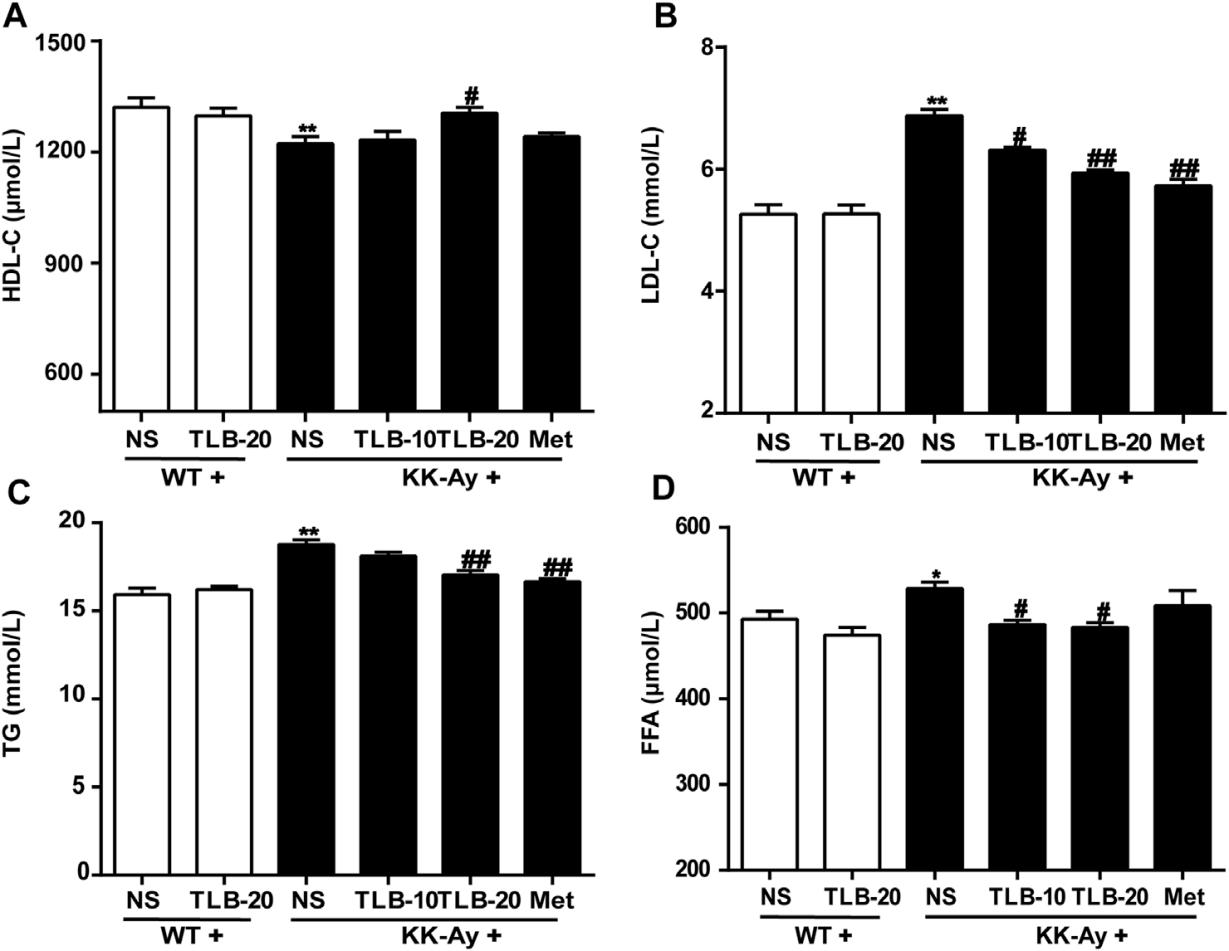
TLB alleviated lipid metabolism disorder in KK-Ay mice. **(A)** HDL-C content. **(B)** LDL-C content. **(C)** TG content. **(D)** FFA content. ^*^ *p* < .05, ^**^ *p* < .01 *vs.* WT group; ^*#*^ *p* < .05, ^*##*^ *p* < .01 *vs.* KK-Ay group; *n* = 6–9 per group, mean ± SEM.

To evaluate the effect of TLB on the lipid metabolism in KK-Ay mice, the contents of TG, HDL-C, LDL-C, and FFA were detected by ELISA. The results showed that the HDL-C level of KK-Ay mice was significantly decreased, but the levels of LDL-C, TG, and FFA were markedly increased more than those of WT mice. However, TLB significantly reversed these changes in mice of the KK-Ay + TLB-20 group than those of the KK-Ay group. These results suggested that TLB effectively alleviated the disorder of lipid metabolism in KK-Ay mice (Figure 5).

### TLB Mitigated Oxidative Stress in KK-Ay Mice

**FIGURE 6:**
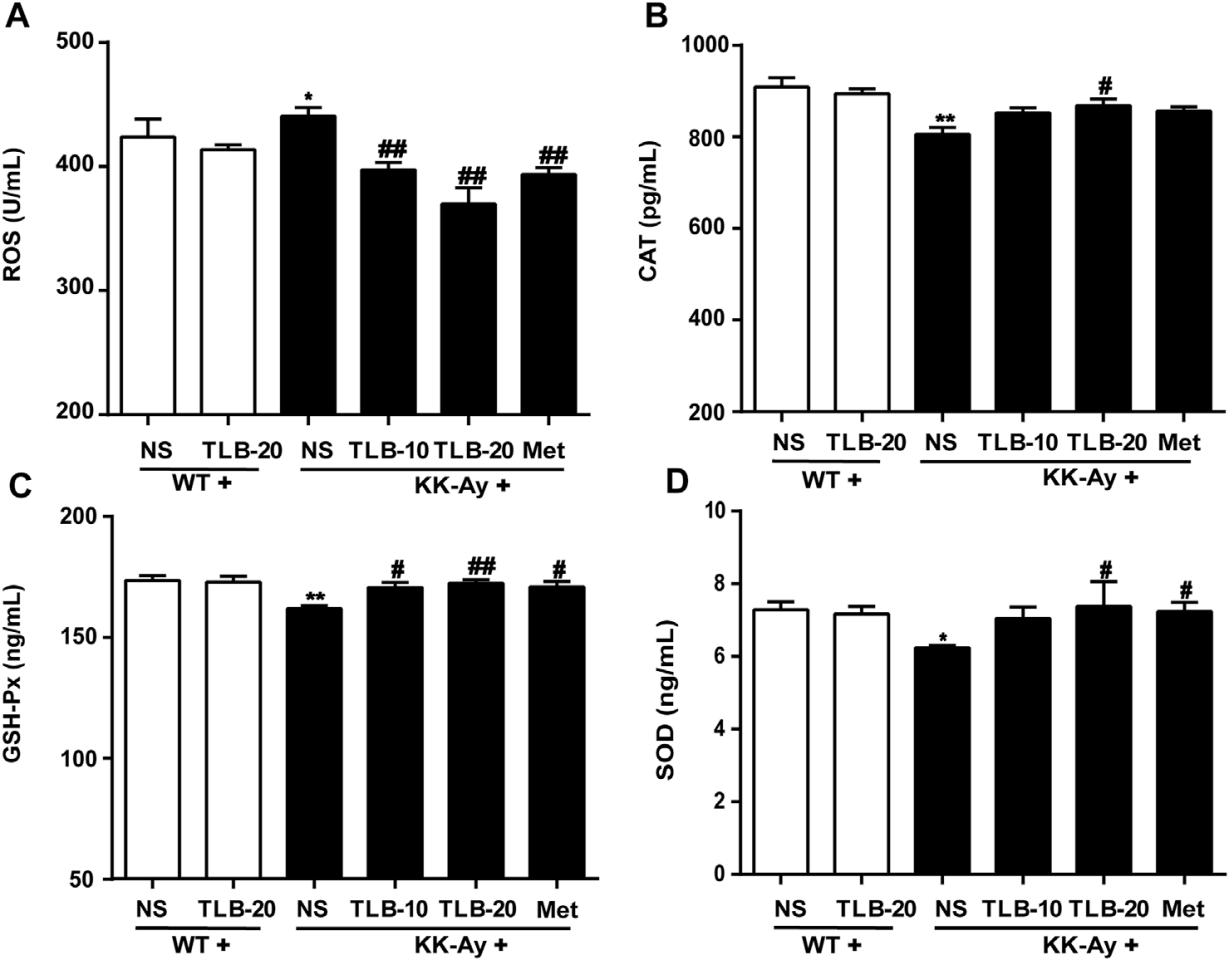
TLB mitigated oxidative stress in KK-Ay mice. **(A)** ROS content. **(B)** CAT activity. **(C)** GSH-Px activity. **(D)** SOD activity. ^*^ *p* < .05; ^**^ *p* < .01 *vs.* WT group; ^*#*^ *p* < 0.05, ^*##*^ *p* < .01 *vs.* KK-Ay group; *n* = 6–9 per group, mean ± SEM.

To assay the effect of TLB on oxidative stress in KK-Ay mice, the content of ROS and activities of CAT, GSH-Px, and SOD were measured using ELISA. The results indicated that the ROS content of KK-Ay mice was significantly increased, while CAT, GSH-Px, and SOD activities were markedly decreased compared to those of WT mice. In contrast, TLB obviously reversed these changes of the KK-Ay + TLB-20 group compared to those of the KK-Ay group. These findings indicated that TLB effectively mitigated oxidative stress of KK-Ay mice (Figure 6).

### TLB Relieved Oxidative Stress by Activating the Nrf2/ARE Signaling Pathway

**FIGURE 7:**
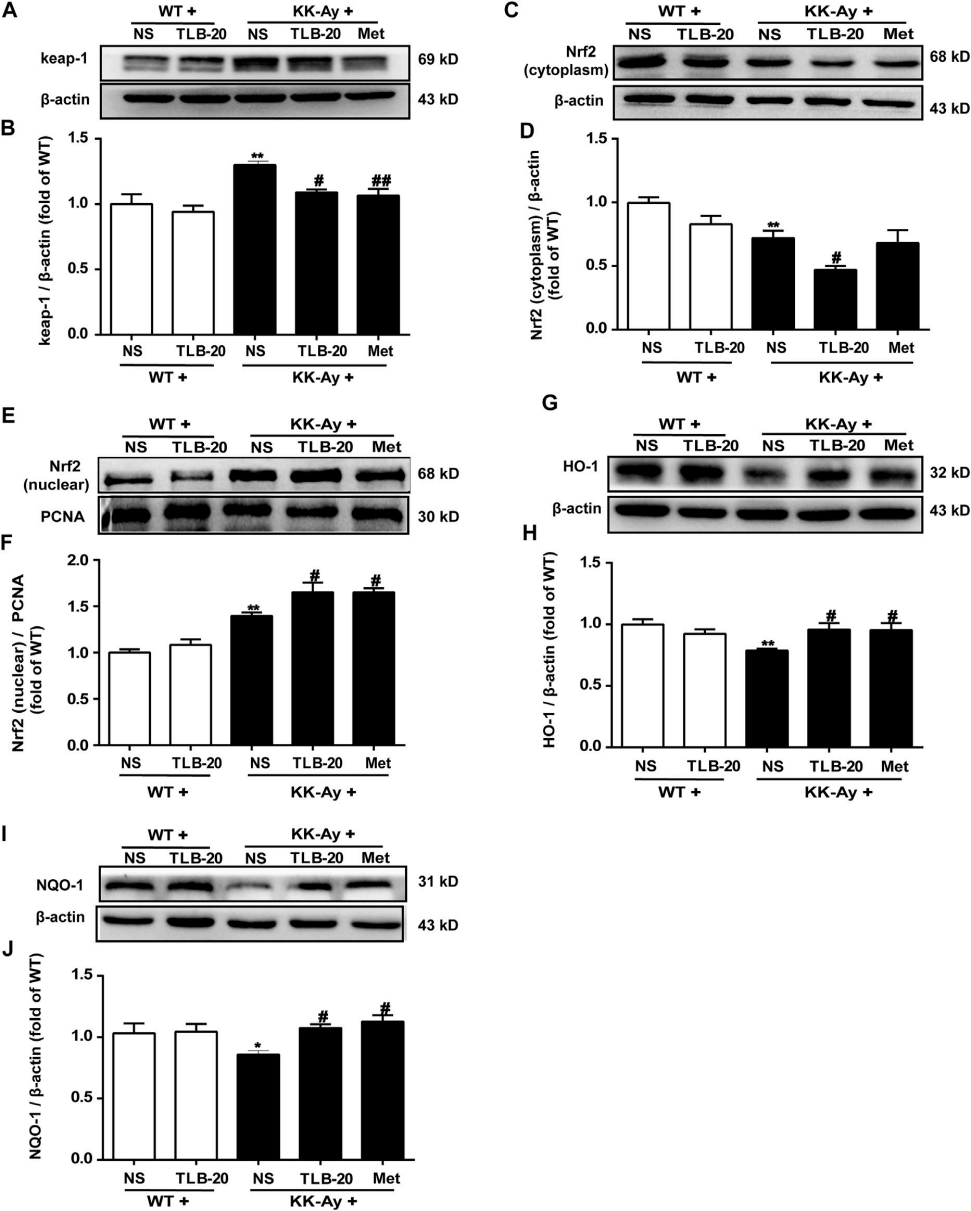
TLB inhibited oxidative stress *via* activating the Nrf2/ARE signaling pathway in the pancreas of KK-Ay mice. **(A)** Representative bands of keap1. **(B)** Quantification of keap1 protein expression. **(C)** Representative bands of cytoplasm Nrf2. **(D)** Quantification of cytoplasm Nrf2 protein expression. **(E)** Representative bands of nuclear Nrf2. **(F)** Quantification of nuclear Nrf2 protein expression. **(G)** Representative bands of HO-1. **(H)** Quantification of HO-1 protein expression. **(I)** Representative bands of NQO-1. **(J)** Quantification of NQO-1 protein expression. ^*^ *p* < .05, ^**^ *p* < .01 *vs.* WT group; ^*#*^ *p* < .05, ^*##*^ *p* < .01 *vs.* KK-Ay group; *n* = 4-5 per group, mean ± SEM.

To further investigate the effect of TLB on the Nrf2/ARE signaling pathway, proteins extracted from the pancreas were determined using western blot. The results showed that the keap1 and the level of nuclear-Nrf2 protein were significantly upregulated while the levels of cytoplasm-Nrf2, HO-1, and NQO-1 expressions were markedly downregulated in the pancreas in the KK-Ay group compared with the WT group. However, TLB significantly reversed these changes in the KK-Ay + TLB-20 group compared with the KK-Ay group. These findings supported that the inhibitory effect of TLB on oxidative stress of KK-Ay mice, at least in part, through the activation of the Nrf2/ARE signaling pathway (Figure 7).

### TLB Attenuated Insulin Resistance in KK-Ay Mice by Regulating the Abnormal Insulin Signaling Transduction Pathway

**FIGURE 8:**
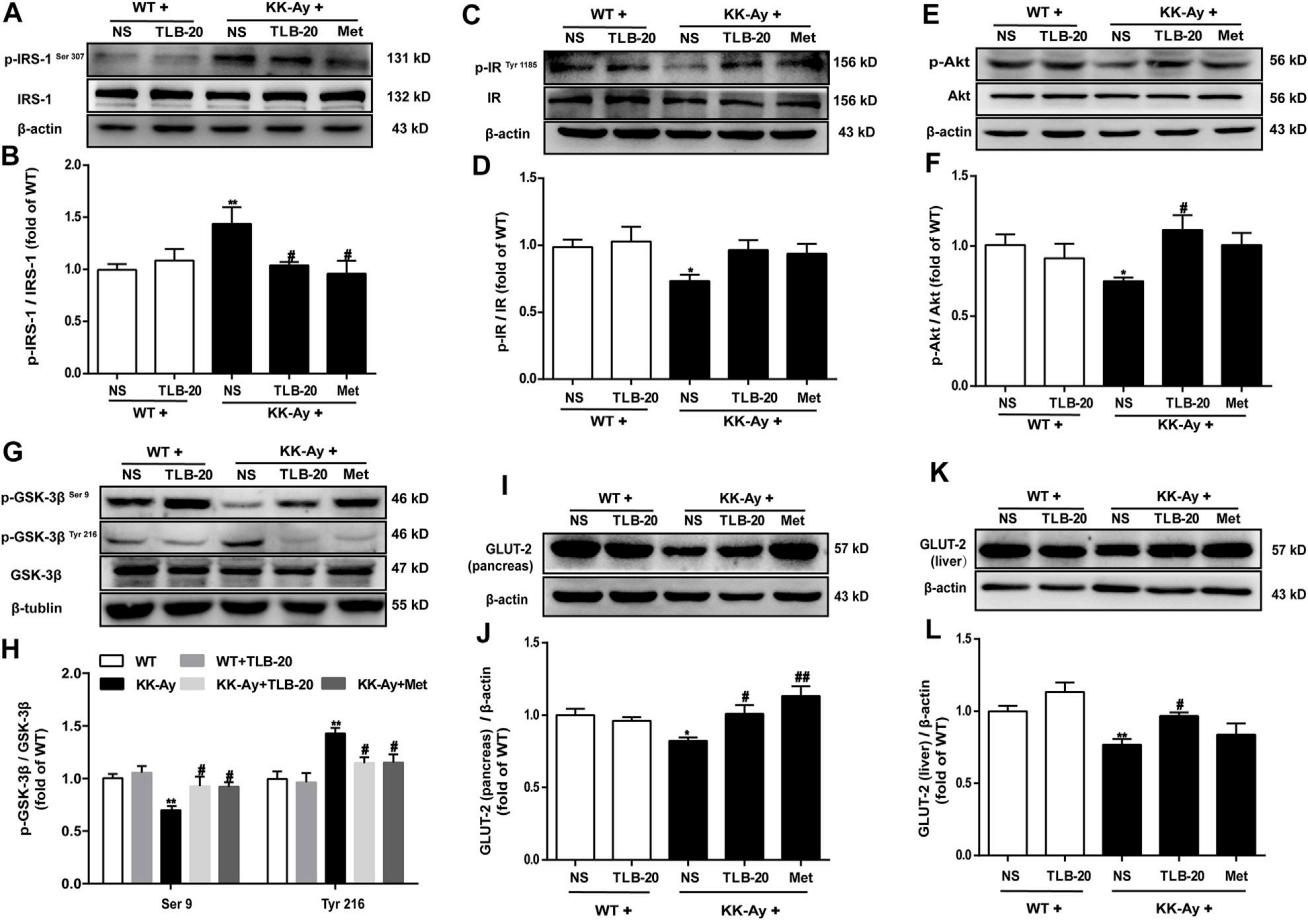
TLB attenuated insulin resistance in KK-Ay mice by regulating abnormal insulin signaling transduction pathway. **(A)** Representative bands of p-IRS-1^Ser 307^ in the pancreas. **(B)** Quantification of p-IRS-1^Ser 307^ phosphorylation level in the pancreas. **(C)** Representative bands of p-IR^Tyr 1185^ in the pancreas. **(D)** Quantification of p-IR^Tyr 1185^ phosphorylation level in the pancreas. **(E)** Representative bands of p-Akt in the pancreas. **(F)** Quantification of p-Akt phosphorylation level in the pancreas. **(G)** Representative bands of p-GSK-3β^Ser 9^ and p-GSK-3β^Tyr 216^ in the pancreas. **(H)** Quantification of p-GSK-3β^Ser 9^ and p-GSK-3β^Tyr 216^ phosphorylation levels in the pancreas. **(I)** Representative bands of GLUT-2 in the pancreas. **(J)** Quantification of GLUT-2 protein expression in the pancreas. **(K)** Representative bands of GLUT-2 in the liver. **(L)** Quantification of GLUT-2 protein expression in the liver. ^*^ *p* < .05, ^**^ *p* < .01 *vs.* WT group; ^*#*^ *p* < .05, ^*##*^ *p* < .01 *vs.* KK-Ay group; *n* = 4-5 per group, mean ± SEM.

Because the insulin signaling transduction pathway plays a critical role in insulin resistance, we further elucidated the effect of TLB on the insulin signaling transduction pathway. The results showed that the phosphorylation levels of p-IRS-1^Ser 307^ and p-GSK-3β^Tyr 216^ were significantly upregulated while p-IR^Tyr 1185^, p-Akt, and p-GSK-3β^Ser 9^ were evidently downregulated in the pancreas in the KK-Ay group than those of the WT group. However, TLB significantly reversed the changes of the KK-Ay + TLB-20 group compared to those of the KK-Ay group (Figures 8A–H). Additionally, the protein expressions of GLUT-2 in the pancreas and liver of KK-Ay mice were significantly downregulated compared to that of WT mice, whereas the protein expressions of GLUT-2 in the pancreas and liver were markedly upregulated in mice of the KK-Ay + TLB-20 group compared to those of KK-Ay group (Figure 8I-L). These findings manifested that the underlying mechanism of TLB attenuated insulin resistance in KK-Ay mice might be related to the regulation of the insulin signaling transduction pathway.

## Discussion

**FIGURE 9:**
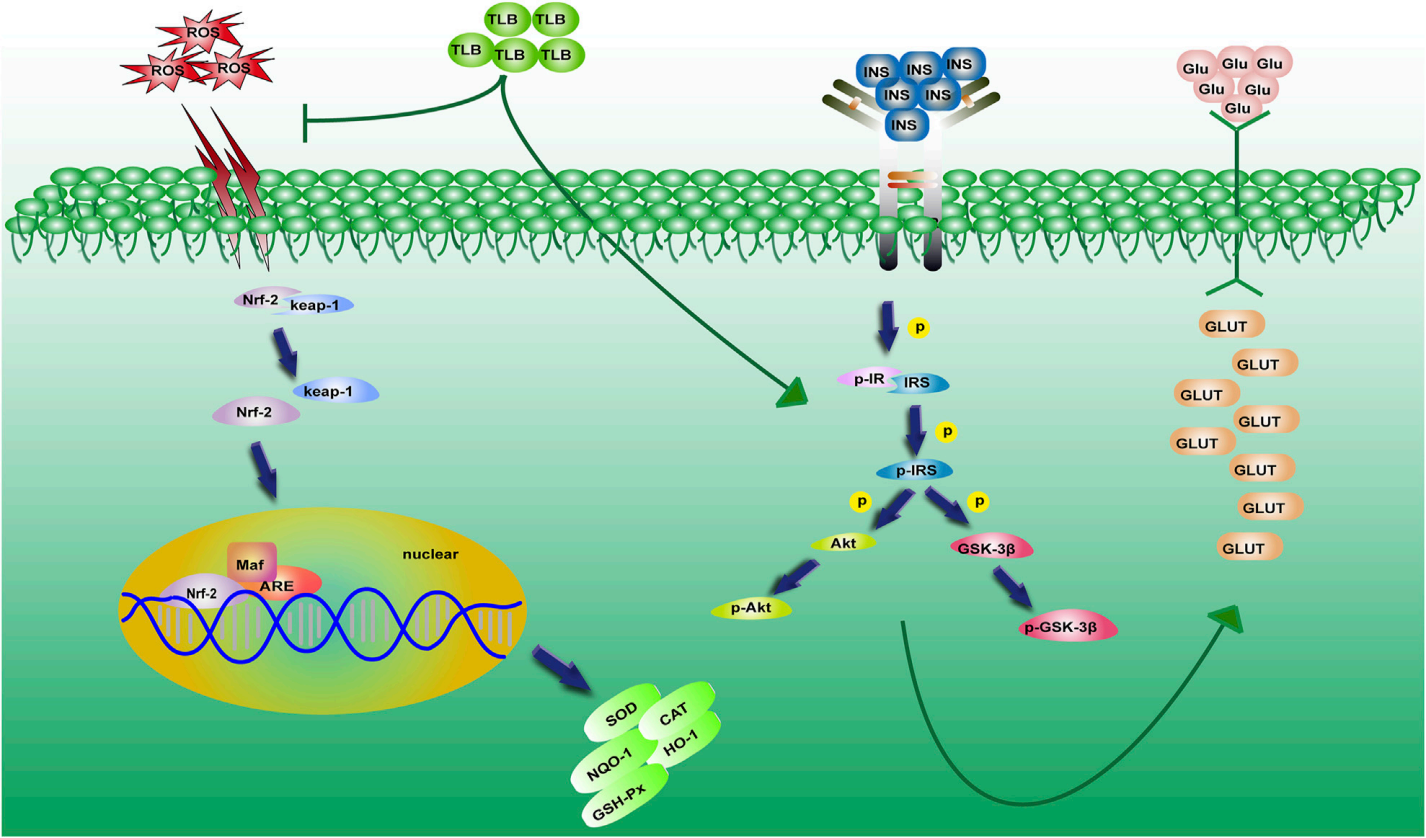
A schematic showing the underlying mechanisms of the anti-T2DM effect of TLB in KK-Ay mice. TLB relieved oxidative stress by activating the Nrf2/ARE signaling pathway and mitigated insulin resistance by regulating the insulin signaling transduction pathway in KK-Ay mice.

This study demonstrated that 1) TLB, a naturally occurring food additive, effectively mitigated the high glucose level of KK-Ay mice; 2) TLB not only effectively alleviated insulin resistance and lipid metabolism disorder but also enhanced antioxidant capacity in KK-Ay mice; and 3) the anti-T2DM effect of TLB was, at least partly, *via* mediating Nrf2/ARE signaling pathway and insulin signaling transduction pathway (Figure 9).

KK-Ay mice, a kind of model animals, were widely used to develop anti-T2DM drugs or detect the therapeutic targets of T2DM because they are similar to the clinical symptoms of T2DM patients (Kitada et al., 2016). Therefore, KK-Ay mice were applied to explore the effect of TLB on T2DM. In the current study, we found that KK-Ay mice showed a high FBG level with polydipsia, polyphagia, and polyuria, in keeping with the previous reports (Liu et al., 2018). In contrast, TLB significantly reduced the high FBG level of KK-Ay mice, which suggested that TLB exerted a notable hypoglycemic effect and deserved further exploration.

OGTT is a glucose load test to measure the adjusting ability of blood glucose after exogenous glucose infusion, and it is also widely used to diagnose DM in clinic. Under normal physiological conditions, the blood glucose will reach the peak at 0.5–1 h after glucose intake, and then return to FBG level 2 h later; however, patients with T2DM or impaired glucose tolerance show reduced glucose tolerance, high blood glucose levels, or rhythm disturbances (Wang et al., 2020). Hence, in this study, we used OGTT to detect the effect of TLB on glucose tolerance in KK-Ay mice. The results showed that the regulation of blood glucose was impaired in KK-Ay mice, which was consistent with the previous reports (Liu et al., 2017b). However, TLB effectively enhanced the glucose tolerance of KK-Ay mice. Likewise, ITT is implemented to test the body’s sensitivity to exogenous insulin (Da et al., 2020). The results showed that KK-Ay mice had a low sensitivity to exogenous insulin, that is why the mice kept a high level of blood glucose after injection of recombinant human insulin, in keeping with the previous studies (Ogiwara et al., 2018). However, TLB significantly increased the insulin tolerance of KK-Ay mice. These findings suggested that TLB exerted an anti-T2DM effect by improving impaired glucose tolerance and insulin tolerance in KK-Ay mice.

Moreover, with the progressive of T2DM, pancreatic *β* cells become decompensated and show insulin secretory defect, subsequently developing into hypoinsulinemia (Holmes, 2017). Our results showed that the morphology of pancreatic islets in KK-Ay mice appeared anomalous, and the number of cells and the expression of insulin were dramatically decreased, consistent with a previous report (Yang et al., 2015). Of note, TLB significantly improved the islet morphology to normal, increased the number of cells and expression of insulin in the islets, and suggested that TLB exhibited the protection of pancreatic islets and upregulation of insulin secretion. T2DM is also accompanied by dyslipidemia, which greatly enhances the risk of cardiovascular diseases (Athyros et al., 2018). Unsurprisingly, TG, FFA, LDL-C, and HDL-C of KK-Ay mice were abnormal, in keeping with previous reports, indicating that KK-Ay mice showed dyslipidemia (Huang et al., 2016). However, TLB markedly regulated the aberrant lipid metabolism of KK-Ay mice by reversing the change of serum TG, FFA, LDL-C, and HDL-C. Furthermore, insulin resistance refers to the decrease even loss of sensitivity of target tissues to insulin, leading a certain amount of insulin cannot achieve the expected biological effects (Mancusi et al., 2020). As the main pathogenesis of T2DM, insulin resistance runs through the whole disease process, induced by impaired insulin signaling transduction and quantified by a steady-state model evaluation of the HOMA-IR score (Wondmkun, 2020). IR, the initial point of insulin signaling transduction, is activated by insulin and then triggers the phosphorylation of a series of downstream genes such as IRS-1, Akt, and GSK-3*β*. These genes lead to the vesicles containing GLUT-2 in the cytoplasm translocating to the cell membrane and fusing with it, ultimately completing the transmembrane transport of glucose (Posner, 2017). In the present study, KK-Ay mice showed obvious insulin resistance, increased serine phosphorylation levels of IRS-1 and tyrosine phosphorylation of GSK-3*β*, decreased activation of Akt and serine phosphorylation of GSK-3*β*, and protein expression of GLUT-2, in keeping with previous studies. It indicated that the insulin signaling transduction pathway was involved in the insulin resistance of KK-Ay mice (Wu et al., 2021). In contrast, TLB markedly mitigated insulin resistance in KK-Ay mice by reversing the changes of GLUT-2, p-IRS-1, p-Akt, and p-GSK-3*β*. These findings indicated that the anti-T2DM effect of TLB, at least in part, IRS-1/GLUT2 signaling pathway.

Of note, emerging evidence suggests that oxidative stress plays a vital role in the pathogenesis of T2DM (Yaribeygi et al., 2020). Nrf2 is a key transcription factor in regulating oxidative stress responses, and the activation of the Nrf2/ARE signaling pathway is important in resisting oxidative stress (Behl et al., 2021). In general, Nrf2 binds to keap1 in the cytoplasm and can be degraded by ubiquitin in an inactivated status. Upon oxidative stress stimuli, Nrf2 is released from keap1 and translocates from the cytoplasm to the nucleus. After forming a heterodimer with the small Maf protein, Nrf2 recognizes and binds with ARE, initiating the transcription of the downstream related antioxidant enzyme genes (David et al., 2017). Notably, TLB not only downregulated the protein expression of keap1 but also promoted the nuclear translocation of Nrf2 to activate the Nrf2/ARE signaling pathway and then initiated the transcription of downstream antioxidant enzyme genes such as CAT, GSH-Px, SOD, HO-1, and NQO-1 in the pancreatic tissue of KK-Ay mice. These findings suggested that the potential mechanism of TLB against T2DM might be related to the activation of the Nrf2/ARE signaling pathway.

In summary, this study preliminarily revealed that TLB exerts an excellent anti-T2DM effect with high safety and stability profile through the activation of the Nrf2/ARE signaling pathway and the mediation of insulin signaling transduction pathway. These findings indicate that TLB is a promising novel candidate for the treatment of T2DM in clinic due to its favorable druggability.

## Data Availability

The original contributions presented in the study are included in the article/supplementary material. Further inquiries can be directed to the corresponding author.
